# Primary CNS Anaplastic Large Cell Lymphoma DUPS22 Rearranged with Gain of ALK: A Case Report and Review of the Literature

**DOI:** 10.1002/jha2.70324

**Published:** 2026-07-17

**Authors:** Sindu Iska, María Herrán, Zackary Anderson, Pablo Bejarano, Silvia Bunting, Chieh‐Lin Fu

**Affiliations:** ^1^ Department of Hematology and Oncology Cleveland Clinic Florida Hospital Weston Florida USA

**Keywords:** ALK‐negative, anaplastic large cell lymphoma, DUSP22 rearrangement, primary CNS lymphoma

## Abstract

ALK‐negative anaplastic large cell lymphoma (ALCL) is a rare primary central nervous system lymphoma (PCNSL). Although DUSP22 rearrangement is considered favorable in systemic ALK‐negative ALCL, survival remains poor in PCNSL. We report a case of PCNSL ALK‐negative ALCL with DUSP22‐IRF4 rearrangement and an unusual gain of the ALK gene (2p23), complicated by transaminitis, treated with high‐dose methotrexate followed by whole‐brain radiation therapy, achieving durable remission exceeding 3 years. To our knowledge, this is the first reported case in the CNS setting, suggesting that DUSP22 rearrangement and ALK gain may confer a favorable prognosis.

**Trial Registration:**

The authors have confirmed clinical trial registration is not needed for this submission.

## 1 Introduction

T‐cell primary central nervous system lymphoma (PCNSL) constitutes less than 5% of all PCNSL. Anaplastic large cell lymphoma (ALCL) is a rare T‐cell subtype strongly positive for CD30, classified into ALK‐positive and ALK‐negative subtypes. ALK‐positive ALCL is defined by a chromosomal translocation involving the ALK gene on chromosome 2p23 and the nucleophosmin (NPM) gene on chromosome 5q35, resulting in the NPM‐ALK chimeric protein [1, 2, 3, 4]. Systemic ALK‐negative ALCL, representing about 40% of ALCL, is genetically heterogeneous, with two recurrent rearrangements identified. Approximately 30% of ALK‐negative ALCL harbor a rearrangement of the DUSP22 gene on 6p25.3, and 8% have a TP63 gene rearrangement on 3q28. Prognosis varies, with DUSP22 rearrangements associated with more favorable outcomes and TP63 indicating more aggressive disease [1, 2, 5, 6, 7]. However, prognostic data for PCNSL ALK‐negative ALCL remain limited [8, 9]. We present a case of PCNSL ALK‐negative ALCL with DUSP22‐IRF4 rearrangement and an unusual finding of ALK gene gain.

## 2 Case Presentation

A 62‐year‐old male with a history of tobacco use and hypertension presented with slurred speech, left facial weakness, confusion, and headache for a week. He denied visual disturbances, focal changes, or constitutional symptoms. Examination revealed dysarthria, left facial droop, and left pronator drift without other motor or sensory deficits. Initial laboratory results are shown in Table 1.

**TABLE 1:** Results of laboratory workup during admission.

Laboratory tests	Result	Normal value
Complete blood count		
White blood cells	11.1	4.0–11.5 10 × 3/µL
Red blood cells	4.75	3.80–5.50 10 × 6/µL
Hemoglobin	13.9	12.8–17.5 g/dL
Hematocrit	41.2	36.0%–50.0%
Mean cell volume	86.7	80.0–100.0 fL
Platelets	207	140–400 10 × 3/µL
Liver function tests		
AST	24	14–40 U/L
ALT	13	10–54 U/L
Alkaline phosphatase	78.0	38–113 U/L
Total bilirubin	0.5	0.2–1.3 mg/dL
Proteins	7.8	6.3–8.0 g/dL
Albumin	4.4	3.9‐4.9 g/dL
Chemistry tests		
Sodium	142	133–145 mmol/L
Potassium	3.8	3.3–5.1 mmol/L
Chloride	108	99–111 mmol/L
CO2	28	21–32 mmol/L
Anion gap	9.8	6.0–18.0 mmol/L
BUN	17	6–25 mg/dL
Creatinine	1.0	0.5–1.5 mg/dL
Glucose	82	70–105 mg/dL
Calcium	91	8.0–10.5 mg/dL

Abbreviations: ALT, alanine transaminase; AST, aspartate transaminase; BUN, blood urea nitrogen.

Brain MRI confirmed a 4.1 × 3.2 cm lesion with lobulated and irregular margins, bright enhancement with central nonenhancement suggesting necrosis, surrounding edema, and a 7 mm right‐to‐left subfalcine shift with effacement of the right frontal horn and the right lateral ventricle (Figure 1). He underwent right frontal craniotomy with microsurgical excision. Histology showed confluent sheets of large atypical lymphoid cells infiltrating the brain parenchyma (Figure 2). The cells demonstrated irregular nuclear contour, coarse chromatin, prominent single or multiple nucleoli, and scant cytoplasm. Occasional cells with multilobate nuclei, consistent with “hallmark” cells, were present. Scattered atypical mitotic figures and angiocentric infiltration were noted. Immunohistochemistry showed diffuse strong positivity for CD30, variable positivity for CD3, CD4, and BCL‐2, and focal positivity for CD25. Stains were negative for CD5, CD7, CD8, CD20, CD43, CD56, ALK‐1, and TIA‐1. Ki‐67 showed a 90% proliferative index. Fluorescence in situ hybridization was negative for ALK rearrangement but positive for gain of ALK (2p23), detected in 56% of interphase cells, and positive for DUSP22‐IRF4 rearrangement. Karyotyping revealed no metaphase cells. Bone marrow reported no lymphomatous involvement.

**FIGURE 1:**
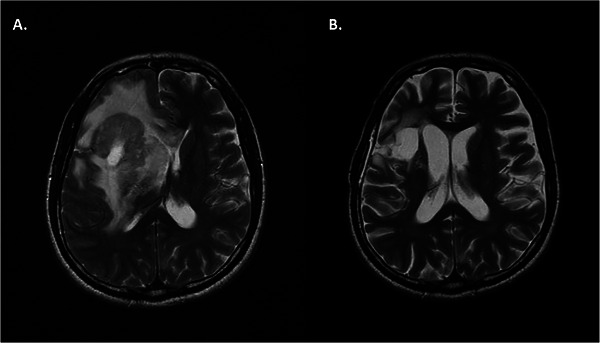
MRI of the brain showing interval changes after resection (axial view). (A) Preoperative MRI demonstrates a 4.1 × 3.2 cm enhancing lesion in the right frontal lobe. (B) MRI at 18 months from diagnosis showing resolution of enhancement along the superior margin of the surgical bed in the right frontal region with no evidence of tumor recurrence.

**FIGURE 2:**
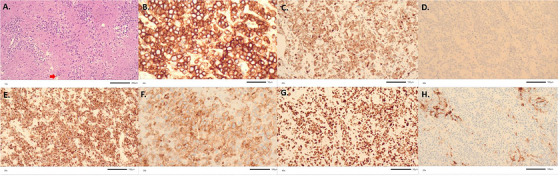
Morphology and immunophenotype of the CNS lesion. (A) H section showing brain parenchyma infiltrated by pleomorphic atypical lymphoid cells with ovoid irregular nuclear contours. Rare “hallmark” cells are present (arrow). The infiltrates were strongly positive for CD30 (B) and CD3 (C) but negative for ALK (D). The infiltrates were positive for CD2 (E) and weakly positive for CD4 (F). Ki‐67 (G) showed a high proliferative index of 95%. The infiltrates were negative for CD56 (H).

During admission, liver function tests became elevated: AST peaked at 238 U/L and ALT at 676 U/L, with normal total bilirubin and alkaline phosphatase. Testing for viral etiologies, including hepatitis A, B, and C, HIV, EBV, and HTLV‐1, was negative. As PET/CT could not be performed during hospitalization, CT scans were used for staging, which was deemed appropriate per NCCN guidelines [10]. CT scan of the chest showed nonspecific atelectasis. CT scan of the abdomen and pelvis with contrast revealed fatty liver without suspicious lesions. MRI of the liver confirmed diffuse hepatic steatosis with two hepatic hemangiomas. Percutaneous liver biopsy showed severe steatosis (80%) with tissue eosinophilia in portal tracts mixed with lymphocytes, which was attributed to drug effects without evident lymphoma (Figure 3).

**FIGURE 3:**
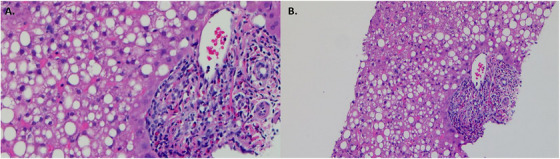
Liver biopsy. (A and B) The liver shows severe steatosis (80%). The portal tract (right) shows numerous eosinophils accompanying lymphocytes, attributed to drug effects without evidence of lymphoma (200× and 100×).

Given his fair performance status, nonalcoholic fatty liver disease, and drug‐induced hepatitis attributed to statin and trimethoprim‐sulfamethoxazole (both discontinued), treatment was initiated with modified single‐agent high‐dose methotrexate (MTX) at 2.5 g/m^2^ with dose escalation to a conventional dose of 3.5 g/m^2^ for 8 cycles. Although MTX can cause liver fibrosis in the setting of underlying fatty liver, high‐dose MTX (> 3 g/m^2^) for 5–8 cycles remains the mainstay of treatment for PCNSL [11, 12]. While ALCL is CD30‐positive, brentuximab vedotin, an anti‐CD30 antibody‐drug conjugate, has limited CNS penetration [13]. A recent case report of refractory primary CNS ALK‐negative ALCL treated with CD30/CD7 CAR‐T cells represents a potential future option [14].

The patient was deemed ineligible for hematopoietic stem cell transplant due to suboptimal diffusing lung capacity (40%) despite optimization. He received whole‐brain radiation therapy (WBRT) to a total dose of 23.4 Gy. MRI imaging at 18 months confirmed continued remission.

## 3 Discussion

Age and ALK status are prognostic in PCNSL ALCL [13]. A systematic review by Hirano et al. [9] reported 39 cases of PCNSL ALCL with a 2‐year overall survival rate of 58%, favoring patients with ALK‐positive disease, age ≤ 40, and MTX‐based treatment. The 2‐year survival rates for ALK‐positive and ALK‐negative PCNSL were 71% and 22%, respectively. Among the 13 cases of CNS ALK‐negative ALCL, 10 with outcome data showed the patients died within 4 days to 6 months after surgery. Only 18% of ALK‐negative PCNSL ALCL patients received high‐dose MTX, 13% received WBRT, and the remainder received supportive care due to rapid disease progression [9].

A study of 81 systemic ALCL cases using interphase FISH found extra copies of the ALK gene locus in 38% of ALK‐positive and 48% of ALK‐negative cases. Cases with extra ALK copies had significantly longer mean survival than those without (64.4 vs. 35.3 months). This favorable effect was observed in both the ALK‐positive (72.3 vs. 45.9 months) and ALK‐negative (34.7 vs. 9.9 months) subgroups. Multivariate analysis confirmed that both ALK protein expression and extra copies of the ALK gene were independent predictors of better survival (*p* = 0.008) [15]. However, the combined effect of these molecular features in PCNSL ALCL remains undefined.

This case has several strengths: it is the first to document the co‐occurrence of DUSP22‐IRF4 rearrangement and ALK gene gain in PCNSL ALCL, and it demonstrates a durable response exceeding 3 years in a disease with historically dismal outcomes. Limitations include the inherent constraints of a single case report, which preclude definitive conclusions regarding the prognostic significance of this molecular combination.

## 4 Conclusion

To our knowledge, this is the first report of ALK‐negative primary CNS ALCL with DUSP22‐IRF4 rearrangement and concurrent ALK gene gain (2p23). Despite initial transient transaminitis, treatment with dose‐escalated high‐dose MTX followed by WBRT achieved a durable remission exceeding 3 years. The combination of DUSP22 rearrangement and ALK gain may contribute to the favorable outcome observed, though this remains to be validated in larger studies.

## Author Contributions

S.I. is a major contributor to writing the manuscript. M.H. participated in writing and major editing of the manuscript with figure design. Z.A. participated in writing and editing the manuscript. P.B. and S.B. provided the histologic images and their interpretation. C.F. is a major contributor to the design and writing of the manuscript. All authors read and approved the final manuscript.

## Funding

The authors have nothing to report.

## Consent

Consent was obtained from the patient.

## Conflicts of Interest

The authors declare no conflicts of interest.

## Data Availability

The data that support the findings of this study are available from the electronic medical records of the patient. Data are available from the authors upon reasonable request and with permission of the patient.
